# Supplementary material for: Identification of photoperiod-induced specific miRNAs in the adrenal glands of Sunite sheep (Ovis aries)
Source: Front Vet Sci. 2022 Jul 22;9:888207. doi: 10.3389/fvets.2022.888207 (PMC9354845; doi:10.3389/fvets.2022.888207)
Supplement: Supplementary file 1 [file Data_Sheet_1.ZIP › Supplementary file/miRDeep2 file.zip!/miRDeep2 file/Know_Structure/SPLP42A1/oar-mir-29b-2.pdf]

miRBase precursor : oar-mir-29b-2
Total read count : 10935
oar-miR-29b read count : 10131
remaining reads : 804

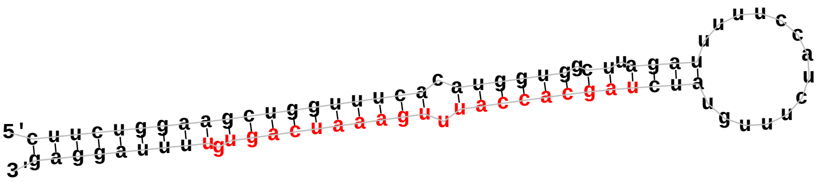

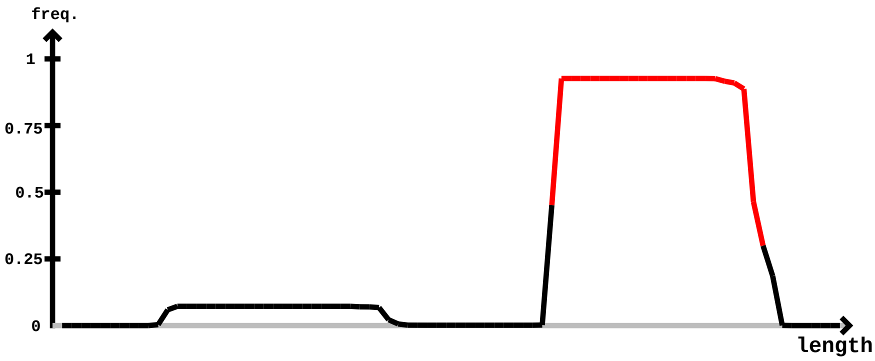

oar-miR-29b

```
5'- cuucuggaagcugguuucacaugguggcuuagauuuuuccaucuuuguaucuagcaccauuugaaaucaguguuuuaggag   -3'   exp
    (((((((((((((((((((.((((((.((.((((..............)))))))))))).)))))))))).)))))))))    reads   mm   sample
    .........gcugguuucacaugguggcu....................................................    1       0    seq
    .........gcugguuucacaugguggcuu...................................................    5       0    seq
    .........gcugguuucacaugguggcuua..................................................    1       0    seq
    .........gcugguuucacaugguggcuuag.................................................    3       0    seq
    .........gcugguuucacaugguggcuuaga................................................    27      0    seq
    .........gcugguuucacaugguggcuuagauuu.............................................    1       0    seq
    ..........cugguuucacaugguggcuu...................................................    13      0    seq
    ..........cugguuucacaugguggcuua..................................................    2       0    seq
    ..........cugguuucacaugguggcuuag.................................................    14      0    seq
    ..........cugguuucacaugguggcuuaga................................................    433     0    seq
    ..........cugguuucacaugguggcuuagau...............................................    114     0    seq
    ..........cugguuucacaugguggcuuagauu..............................................    29      0    seq
    ..........cugguuucacaugguggcuuagauuu.............................................    1       0    seq
    ..........cugguuucacaugguggcuuagauuuu............................................    1       0    seq
    ...........ugguuucacaugguggcuu...................................................    2       0    seq
    ...........ugguuucacaugguggcuua..................................................    1       0    seq
    ...........ugguuucacaugguggcuuag.................................................    10      0    seq
    ...........ugguuucacaugguggcuuaga................................................    53      0    seq
    ...........ugguuucacaugguggcuuagau...............................................    59      0    seq
    ...........ugguuucacaugguggcuuagauu..............................................    20      0    seq
    .................................uuuuucUaucuuuguauc..............................    1       1    seq
    .................................uuuuuccaucuuuguauU..............................    1       1    seq
    .................................uuuuuccaucuuuguauc..............................    10      0    seq
    .................................uuuuuccaucuuuguaucu.............................    1       0    seq
    ..................................uuuuccaucuuuguauU..............................    1       1    seq
    .................................................ucuagcaccauuugaaaucagu..........    8       0    seq
    ..................................................cuagcaccauuugaaau..............    5       0    seq
    ..................................................cuagcaccauuugaaauc.............    49      0    seq
    ..................................................cuaCcaccauuugaaauc.............    2       1    seq
    ..................................................cuagcaccauuugaaauca............    21      0    seq
    ..................................................cuagcaccauuugaaaucG............    2       1    seq
    ..................................................cuagcaccauuugaaaucag...........    43      0    seq
    ..................................................cuagcaccauuugaaaucGg...........    1       1    seq
    ..................................................cuagcaccauuugaaaucGgu..........    19      1    seq
    ..................................................cuagcaccauuugaaaucagu..........    2574    0    seq
```

oar-miR-29b

```
cuucuggaagcugguuucacaugguggcuuagauuuuuccaucuuuguaucuagcaccauuugaaaucaguguuuuaggag
..................................................cuagcaccauuugaaaucagug.........    1341    0    seq
..................................................cuagcaccauuugaaaucagugu........    857     0    seq
..................................................cuagcaccauuugaaaucaguguu.......    5       0    seq
...................................................uagcaccauuugaaauA.............    1       1    seq
...................................................uagcaccauuugaaauc.............    51      0    seq
...................................................uagcaccauuugaaauU.............    4       1    seq
...................................................uagcaccauuugaaauca............    20      0    seq
...................................................uagcaccauuugaaaucG............    28      1    seq
...................................................uagcaccauuugaaaucag...........    55      0    seq
...................................................uagcaccauuugaaaucGg...........    146     1    seq
...................................................uagcaccauuugaaaucagu..........    1444    0    seq
...................................................uagcaccauuugaaaucGgu..........    582     1    seq
...................................................uagcaccauuugaaaucagug.........    460     0    seq
...................................................uagcaccauuugaaaucagugu........    384     0    seq
...................................................uagcaccauuugaaaucaguguu.......    2020    0    seq
...................................................uagcaccauuugaaaucaguguuC......    2       1    seq
...................................................uagcaccauuugaaaucaguguuu......    5       0    seq
....................................................agcaccauuugaaaucagu..........    1       0    seq
....................................................agcaccauuugaaaucagugu........    1       0    seq
```
